# First Characterization of Chicken Interleukin-9

**DOI:** 10.3389/fimmu.2022.889991

**Published:** 2022-06-20

**Authors:** Shuangjiang He, Lina Chen, Xiaoli Hao, Yi Yang, Shaobin Shang

**Affiliations:** ^1^ College of Veterinary Medicine, Yangzhou University, Yangzhou, China; ^2^ Institute of Comparative Medicine, Yangzhou University, Yangzhou, China; ^3^ Jiangsu Co-innovation Center for Prevention and Control of Important Animal Infectious Diseases and Zoonosis, Yangzhou University, Yangzhou, China; ^4^ International Corporation Laboratory of Agriculture and Agricultural Products Safety, Yangzhou University, Yangzhou, China

**Keywords:** chicken interleukin-9, biological activity, phylogenetic analysis, monoclonal antibodies, tissue distribution

## Abstract

Interleukin-9 (IL-9) is a pleiotropic cytokine that acts on a variety of cells and tissues, and plays roles in inflammation and infection as well as tumor immunity. While mammalian IL-9s have been widely investigated, avian IL-9 has not yet been identified and characterized. In this study, we cloned chicken IL-9 (chIL-9) and performed a phylogenetic analysis, examined its tissue distribution, characterized the biological functions of recombinant chIL-9 (rchIL-9) and the expression form of natural chIL-9. Phylogenetic analysis showed that chIL-9 has less than 30% amino acid identity with mammalian IL-9s. The chIL-9 mRNA can be abundantly detected only in the testis and thymus, and are significantly up-regulated in peripheral blood mononuclear cells (PBMCs) upon mitogen stimulation. The rchIL-9 was produced by prokaryotic and eukaryotic expression systems and showed biological activity in activating monocytes/macrophages to produce inflammatory cytokines and promoting the proliferation of CD3^+^ T cells. In addition, four monoclonal antibodies (mAbs) and rabbit polyclonal antibody (pAb) against rchIL-9 were generated. Using anti-chIL-9 mAbs and pAb, natural chIL-9 expressed by the activated PBMCs of chickens with a molecular weight of 25kD was identified by Western-blotting. Collectively, our study reveals for the first time the presence of functional IL-9 in birds and lays the ground for further investigating the roles of chIL-9 in diseases and immunity.

## Introduction

Interleukin-9 (IL-9) is a pleiotropic cytokine of the common γ-chain receptor cytokine family, with other members including IL-2, IL-4, IL-7, IL-15, and IL-21 (1). It was initially identified as a T cell growth factor and thought to be a type 2 helper T cell (Th2)-specific cytokine (2, 3) but later was shown to be predominantly produced by a distinct subset of T helper cells, namely Th9 cells (4). Besides Th9 cells, IL-9 was found to be secreted by a variety of immune cells including Th17 cells, regulatory T cells (Tregs), cytotoxic CD8 T cells, natural killer T cells (NKT), mast cells, eosinophils, and innate lymphoid cell type 2 (1, 2, 5). IL-9 is biologically active on various cell types from the hemopoietic and lymphoid systems including mast cells, B cells, T cells, hemopoietic progenitors, immature neuronal cell lines, and macrophages and dendritic cells, with different effects (5, 6). For instance, IL-9 was shown to induce T cell proliferation (7) and upregulate the expression of CD86 and MHC class II on the surface of bone marrow-derived dendritic cells and their expression of inflammatory cytokines (8).

IL-9 plays multi-faceted roles in inflammation, infection, and tumor immunity. IL-9 has been reported to induce the development of autoimmune diseases such as inflammatory bowel diseases, multiple sclerosis, rheumatoid arthritis, and so on (5, 9), and to contribute to the pathophysiology of allergic diseases like food allergy and asthma (5). In the context of cancer, IL-9 has shown pro-tumor effects on hematological tumors and solid tumors (10). However, in melanoma, IL-9 and Th9 cells have also shown anti-tumor activity (11, 12). In addition, IL-9 has been shown to modulate immune responses during respiratory syncytial virus infection (13), and play protective roles against *Helicobacter pylori* and parasitic worm infections (14, 15).

In human and mouse, the *Il9* gene encodes a 14-kD glycoprotein composed of 144 amino acids (aa), with a typical signal peptide of 18aa (2). However, natural IL-9 protein was found to be highly glycosylated with molecular weight between 32 and 39 kD (7). Besides its discovery in human and mouse, *Il9* genes in other species have not yet been characterized. Although chicken *Il9* gene is annotated in the genome (16), chicken IL-9 (chIL-9) has not been identified and characterized functionally.

In this study, we did a comprehensive characterization of chIL-9 through gene cloning, phylogenetic analysis, tissue distribution, and functional test of recombinant chIL-9. In addition, by generating monoclonal antibodies (mAbs) and rabbit polyclonal antibody (pAb) against chIL-9, we identified the natural expression form of chIL-9. We found that chIL-9 has low amino acid identity with mammalian IL-9s and is abundantly detectable only in testis and thymus of chickens. The recombinant chIL-9 (rchIL-9) showed biological activity in activating monocytes/macrophages and promoting the proliferation of CD3^+^ T cells. Using anti-chIL-9 mAbs and pAb, we identified natural chIL-9 expressed by activated chicken PBMCs as a glycosylated protein. Our data demonstrated the presence of functional IL-9 in birds.

## Materials and Methods

### Animals, Cell Lines and Antibodies

6-week-old specific-pathogen-free (SPF) White Leghorn chickens were purchased from Zhejiang Lihua Agricultural Technology Co., Ltd. (Ningbo, China). New Zealand White Rabbit, BALB/c and ICR mice were purchased from Comparative Medicine Center of Yangzhou University. SP2/0 myeloma cell, chicken fibroblast cell line DF-1 and macrophage cell line HD11 were gifted by Dr. Aijian Qin and Dr. Jianzhong Zhu at Yangzhou University. Anti-chicken CD3 (CT-3) and CD8α (CT-8) antibodies, conjugated with PerCP-Cy5.5 and AF700 respectively, were purchased from SouthernBiotech (Birmingham, AL, USA). Recombinant chicken IL-2 (rchIL-2) was obtained from Kingfisher (London, UK).

### Isolation of Peripheral Blood Mononuclear Cells, mRNA Extraction and Gene Cloning

Peripheral blood mononuclear cells (PBMCs) of chickens were isolated with a separation kit for chicken PBMCs (TBD, Tianjin, China). Briefly, 5 mL peripheral blood from a chicken with anticoagulant was taken and diluted equally with sample diluent, and then overlaid onto the PBMCs separation solution and centrifuged at 500 g for 30 min at room temperature (RT). The cells at the interface were harvested, washed, and then resuspended in complete RPMI1640 medium containing 5% FBS, 5% chicken serum (Gibco, Grand Island, NY, USA), penicillin (100U/ml) and streptomycin (0.1mg/ml) (Beyotime, Shanghai, China).

The isolated chicken PBMCs were plated in 24-well plate with each well containing 10×10^6^ cells in 1 mL complete RPMI1640 medium and stimulated with phorbol myristate acetate (PMA) and ionomycin at a final concentration of 50 ng and 500 ng per ml, respectively for 4 h. The activated PBMCs were then harvested for mRNA extraction and cDNA reverse transcription. Total RNA was extracted with FastPure Cell/Tissue Total RNA Isolation Kit (Vazyme, Nanjing, China) and reverse transcribed into cDNA with HiScript III RT SuperMix for qPCR (Vazyme, Nanjing, China) according to the manufacturer’s instruction. A pair of primers based on the *Il9* gene of chicken (Genbank accession num: AM773755.1) were designed: Forward, 5’- CCGGAATTC(*EcoRI*)ATGAATGCCAGCATGCTG-3’; Reverse:5’- CGGGATCC(*BamHI*)TTAAACTCTAGATTTATG-3’. Finally, the amplified polymerase chain reaction (PCR) product was cloned into a pMD19-T vector (TaKaRa, Shiga, Japan) and confirmed by DNA sequencing.

### Sequence Characteristics and Phylogenetic Analysis

Chicken IL-9 nucleotide (GenBank accession: AM773755.1) and protein sequence (GenBank accession: ACY79396.1) were retrieved from NCBI. The amino acid sequence was analyzed using the ExPASy Molecular Biology Server (http://www.expasy.ch/tools/) and the potential N-glycosylation sites were obtained from NetNGlyc 1.0 Server (http://www.cbs.dtu.dk/services/NetNGlyc/). BioEdit software (7.2.1) with CLUSTAL W algorithm was used for amino acid sequence analysis. Maximum likelihood phylogeny was generated using MEGA 6 software (6.06), and bootstrap support for each node was evaluated with 1,000 replicates.

### Quantitative Real-Time PCR

Quantitative real-time PCR was employed to quantify the abundance of chIL-9 mRNA in tissues and the mRNA level of inflammatory cytokines by monocytes/macrophages. The organs of SPF chickens including heart, liver, spleen, lung, kidney, bursa, thymus, and testis were harvested and total RNA of each organ was extracted using FastPure Cell/Tissue Total RNA Isolation Kit, and reversely transcribed into cDNA with HiScript III RT SuperMix for qPCR. Then, the quantitative PCR was performed with ChamQ Universal SYBR qPCR Master Mix (Vazyme, Nanjing, China) in a Light Cycler 480 instrument (Roche, Mannheim, Germany). The expression of ch*Il9 *transcripts was quantified using primer pairs: IL9qF, 5’-CTTGTTCATGTCTTCCCATCC-3’; IL9qR, 5’-CAGAGGTTTCTATCCCGTTGA-3’ as listed in **Table 1**. The chIL-9 mRNA expression relative to housekeeping gene chicken β-actin was calculated by 2^−ΔCT^ method, and the mRNA was considered undetectable when there was no amplification curve. In order to quantify chIL-9 mRNA in activated PBMCs, 20×10^6^ PBMCs in 1 mL complete RPMI1640 medium were plated in 24-well plate, and stimulated with or without PMA (50 ng/mL) and ionomycin (500 ng/mL), respectively for 4 h. The cells were then collected for mRNA extraction and cDNA reverse transcription. Lastly, the quantitative PCR for chIL-9 was performed according to the above protocol, and the chIL-9 mRNA expression relative to β-actin was normalized by 2^−ΔΔCT^.

**Table 1:** Primers sequences for real-time PCR.

Target gene	Primer name	Primer sequence (5’-3’)	Accession number
IL-9	IL-9 forward	CTTGTTCATGTCTTCCCATCC	GU119893.1
	IL-9 reverse	CAGAGGTTTCTATCCCGTTGA	
IL-1β	IL-1β forward	CCGAGGAGCAGGGACTTT	DQ393267.1
	IL-1β reverse	AGGACTGTGAGCGGGTGTAG	
IL-6	IL-6 forward	AATCCCTCCTCGCCAATCT	NM_204628.1
	IL-6 reverse	CCCTCACGGTCTTCTCCAA	
iNOS	iNOS forward	CACTACCTGCCTGGAGAACAT	D85422.1
	iNOS reverse	CTTGCCCAATAGCCACCTT	
β-actin	β-actin forward	GATTGGAGGCTCTATCCTGG	L08165.1
	β-actin reverse	TTAGAAGCATTTGCGGTGG	

### Recombinant Expression of chIL-9 in *E.coli*

In order to improve the efficiency of prokaryotic expression, the codons of ch*Il9* gene were optimized for *E. coli*. Truncated ch*Il9* gene without signal peptide sequence was cloned into pET-32a plasmid using a pair of primers (Forward:5’- CGGGATCC(*BamHI*) GAGAACCTGTACTTCCAAGGGCAGAATTGCCAGGTT-3’ (containing TEV enzyme cleavage site) and Reverse:5’-CCGCTCGAG(*XhoI*)TCACACGCGGCTTTTAT-3’ and confirmed by enzymatic digestion and DNA sequencing. The recombinant pET-32a-chIL-9 vector was transformed into *E.coli* ROSETTA (DE3) strain. After induction for 4 h with isopropyl-beta-D-thiogalactopyranoside (IPTG) (Solarbio, Beijing, China) at a final concentration of 0.5 mM, 5mL culture of bacteria were collected and lysed by sonication. The lysates were analyzed by 12% sodium dodecyl sulfate polyacrylamide gel electrophoresis (SDS-PAGE) and stained with Coomassie Blue (Beyotime, Shanghai, China). In order to prepare adequate amount of rchIL-9, 200 mL cell culture was induced at the above conditions. Then, the cell pellets were collected and sonicated, and the lysed precipitate were dissolved with 8M urea. The rchIL-9 protein with Trx-his tag was purified with the Ni Sepharose Column (General Electric, Boston, MA, USA) under denature condition according to the manufacturer’s protocol. The purified rchIL-9 was refolded by dialysis with gradient urea buffer (6M, 4M, 2M, 1M, 0M), and the refolded rchIL-9 was finally quantified by BCA Protein Assay Kit (Beyotime, Shanghai, China). In addition, the Trx-his tag in the rchIL-9 fusion protein was removed by cleavage with Tev enzyme (Beyotime, Shanghai, China) at 4°C overnight, and the rchIL-9 without Trx-his tag was yielded by passing through the Ni Sepharose Column again.

### Transfection and Immunocytochemistry

The ch*Il9* gene from T-vector was transferred into pEGFP-C plasmid by PCR with following primers: Forward:5’-CCGGAATTC(*EcoRI*)GGAAGCGGAGAGGGCAGAGGAAGTCTGCTAACATGCGGTGACGTCGAGGAGAATCCTGGACCTATGAATGCCAGCATGCTG-3’ (containing a T2A peptide) and Reverse:5’-CGGGATCC(*BamHI*)TTAAACTCTAGATTTATG-3’. The ultrapure plasmid pEGFP-C and pEGFP-chIL-9 were extracted using QIAGEN Plasmid Midi Kit (QIAGEN, Frankfurt, Germany) and transfected into DF-1 cells with Lipofectamine™ 2000 Transfection Reagent (Invitrogen, Carlsbad, CA, USA) according to the manufacturer’s protocol. Briefly, DF-1 cells (1×10^4^/well) were seeded in a 96-well flat bottom plate and grown to 70-90% confluence. The cells were transfected with plasmid pEGFP-C or pEGFP-chIL-9 (100 ng/well) along with Lipofectamine^®^ Reagent (0.2 μL/well). Forty-eight hours after transfection, culture supernatant from pEGFP-C and pEGFP-chIL-9-tranfected DF-1 cells was collected for subsequent functional experiments.

For immunocytochemistry (ICC), the transfected cells were fixed with ice cold acetone-ethanol mixture (3:2) for 5 min at RT. The cells were then blocked with 1% bovine serum albumin (BSA) (Beyotime, Shanghai, China) in phosphate buffered saline (PBS) for 1 h at RT. Afterwards, the cells were washed and incubated with negative serum separated from non-immunized BALB/c mice, polyclonal antibody serum (anti-chIL-9 pAb) from chIL-9-immune BALB/c mice, negative serum (Control) and chIL-9 mAbs overnight at 4°C, respectively. After three washed with PBS, the cells were incubated with horseradish peroxidase (HRP)-conjugated anti-mouse IgG (CWBIO, Taizhou, China) for 1 h at RT and washed again. Finally, staining was developed with AEC substrate (Solarbio, Beijing, China) for 15 min, stopped with ultrapure water, and observed under the inverted microscope (NIKON, Shanghai, China).

In order to detect the expression of rchIL-9 by the transfected DF-1 cells using anti-chIL-9 pAb by Western blot. DF-1 cells (60×10^4^/well) were seeded in 6-well plate and grown to 70-90% confluence. The cells were transfected with plasmid pEGFP-C or pEGFP-chIL-9 (2.5 μg/well) along with Lipofectamine^®^ Reagent (6 μL/well). Forty-eight hours after transfection, the cells were collected and lysed in 100 μL Radio Immunoprecipitation Assay (RIPA) lysis buffer (NCM Biotech, Suzhou, China). The cell lysate was centrifuged by 12000g at 4°C for 30min and the supernatant was subjected to 12% SDS-PAGE and Western blot.

### Functional Assay of rchIL-9 on Monocytes/Macrophages

Primary monocytes/macrophages were isolated by the adherence of PBMCs to plate. Briefly, PBMCs (3×10^7^/mL) isolated from chickens were seeded in a 24-well plate and incubated for 2-3 h at 41°C. The adherent monocytes/macrophages were treated for 6 h with the supernatants (1:1 dilution) from pEGFP-transfected (Control), pEGFP-chIL-9-transfected DF-1 cells (rchIL-9) and LPS (100 ng/mL) (as a positive control), respectively. Similarly, chicken macrophage cell line HD11 was plated in 24-well plate (1×10^6^ cells/well) and treated for 6 h with the above condition. After treatment, total RNA of monocytes and HD11 cells was extracted and reversely transcribed into cDNA for qPCR detection of inflammatory cytokines interleukin-1β (IL-1β), interleukin-6 (IL-6), and inducible nitric oxide synthase (iNOS) as the above-mentioned protocol. The relative mRNA expression to β-actin was normalized by 2^−ΔΔCT^ method. The primers for each target gene used for qPCR are summarized in **Table 1**.

### CFSE Labelling, Lymphocyte Proliferation Assay and Flow Cytometry

The isolated PBMCs were firstly labelled with CFSE (BioLegend, San Diego, CA, USA) according to previous report (17). Briefly, 40×10^6^ PBMCs were washed twice with PBS and resuspended in 1 ml PBS, then 1 μl CFSE was added at a final concentration of 5 μM and incubated for 10 min in the dark at RT. Thereafter, an equal volume of FBS was added and sit for 1 min. After washing twice with PBS, the CFSE-labelled PBMCs were seeded into a 96-well round bottom plate (2×10^6^ cell/well) and cultured for 5 days in the presence of the supernatant from pEGFP-C-transfected DF-1 cells (Control), pEGFP-chIL-9-transfected DF-1 cells diluted at 1:1 ratio, purified rchIL-9 (100 ng/mL), and rchIL-2 (100 ng/mL), respectively. Thereafter, the cells were harvested and stained with fixable viability dye (FVD) eFluor 780 (Thermo Fisher Scientific, Waltham, MA, USA) for excluding dead cells, followed by staining with 50 μl cocktail containing anti-chicken CD3 and CD8α antibodies for 20 min at RT as previously reported (18). The cells were then washed once and resuspended with FACS buffer for flow cytometric analysis. Flow cytometry was performed with a FACS LSRFortessa (BD Biosciences, Franklin Lakes, NJ, USA). A minimal number of 100,000 cells was acquired and the data were analyzed by FlowJo software (Tree Star Inc., Ashland, OR, USA).

### The Generation of Monoclonal Antibodies Against chIL-9

The rchIL-9 produced from *E. coli* was used as an immunogen to generate anti-chIL-9 mAbs following our previous protocol (19). 6-week-old BALB/c mice were immunized for three times at two weeks interval with 40 μg of rchIL-9 in Freund’s adjuvant (Sigma-Aldrich, Poole, UK) by intraperitoneal injection. Three days after last boost immunization with 80 μg of rchIL-9. Hybridomas were obtained by the fusion of splenocytes of immunized mice with SP2/0 cells in the presence of PEG1500 (Roche, Mannheim, Germany) at 37°C, and hybridomas were selected in DMEM complete culture medium (Gibco, Grand Island, NY, USA) containing hypoxanthine, aminopterin and thymidine (HAT) (Thermo Fisher Scientific, Waltham, MA, USA) for 10 days. Hybridomas secreting anti-chIL-9 mAbs were screened by ELISA, in which rchIL-9 produced from *E. coli* was coated as antigen. Positive hybridomas were cloned twice by limiting dilution methods. The resulting positive hybridomas were further characterized by immunohistochemistry for its reactivity with eukaryotic rchIL-9 expressed in DF-1 cells as aforementioned procedure and their IgG subclass and light chain class were determined with the Mouse Immunologlobulin Isotyping ELISA Kit (BD Biosciences, Franklin Lakes, NJ, USA) according to the manufacturer’s instruction. Mouse ascites containing anti-chIL-9 mAbs were produced by injecting the hybridomas into the abdominal cavity of BALB/c mice that were pre-injected with pristane 7 days ago (Sigma-Aldrich, Poole, UK). The mAb in the ascites was purified with Protein A+G Agarose (Beyotime, Shanghai, China).

### Enzyme-Linked Immunosorbent Assay

Ninety-six-well plates were coated with 100 μl rchIL-9 at a concentration of 1 μg/mL overnight at 4°C, and then blocked with 5% skimmed milk for 2 h at 37°C. After washed with PBS containing 0.5% Tween 20 (PBS-T), hybridoma cell culture supernatant was added and incubated for 2 h at 37°C. After washed five times, HRP-conjugated goat anti-mouse IgG secondary antibody diluted at 1 to 10,000 was added and reacted for 1 h at RT. Following final washing with PBS-T, the color was developed with TMB substrate (Beyotime, Shanghai, China) for 10 min and the plate was read at 450 nm with a microplate reader (Tecan, Switzerland).

### The Production of Rabbit Anti-chIL-9 Polyclonal Antibody

The rchIL-9 produced from *E. coli* was used as an immunogen to generate rabbit anti-chIL-9 polyclonal antibodies (rpAb). 4-month-old New Zealand white rabbits were immunized subcutaneously every two weeks for 3 times with 100 μg rchIL-9 in Freund’s incomplete or complete adjuvants. Seven days after last booster immunization with 200 μg rchIL-9, the anti-chIL-9 serum was separated from blood, and the pAb in the serum was purified with Protein A+G Agarose (Beyotime, Shanghai, China) according to the manufacturer’s instruction.

### Identification of Natural Expression Form of chIL-9 by Western Blot

Chicken PBMCs were isolated and activated by PMA and ionomycin in the presence or absence of brefeldin A (5 μg/mL) (Biolegend, San Diego, CA, USA) as above-mentioned protocol, which allowed cytokine accumulation inside the cells or secretion into the supernatant. The total protein extracted from 20×10^6^ activated PBMCs was obtained in 100 μL RIPA lysis buffer and the culture supernatant was collected. Subsequently, 5x loading buffer was added and boiled for 10 min. Three μL pre-stained protein ladder (Vazyme, Nanjing, China), thirty microgram total protein or 30 μL the supernatant was loaded to each lane and subjected to 12% SDS-PAGE. Then, the gel was transferred to polyvinylidene fluoride (PVDF) membrane (Millipore, MA, USA) (200 mA, 90 min). The PVDF membranes were blocked with 5% skim milk for 1 h at RT and then incubated with indicated anti-chIL-9 mAbs or rpAb overnight at 4°C. After washed 3 times with TBS buffer containing 0.5% Tween 20 (TBS-T), the membranes were treated with HRP-conjugated anti-mouse or anti-rabbit secondary antibody (1:8000) for 1 h at 37 °C. The membranes were washed again with TBS-T, and protein bands were developed with enhanced chemiluminescence substrate (NCM Biotech, Suzhou, China) and visualized with an electrochemiluminescence detection system (Tanon, Shanghai, China).

### Statistical Analysis

Statistical analysis was performed with GraphPad Prism software (GraphPad, La Jolla, CA). When comparing experimental values from two groups, one- or two-tailed student’s t-tests were routinely used. A significant difference compared to the control group was indicated as: *p* < 0.05 (*), *p* < 0.01 (**), and *p* < 0.001 (***).

## Results

### Sequence Characteristics and Phylogenetic Analysis of Chicken IL-9

In line with the annotation in the genome of chicken, the cloned chicken *Il9* gene consists of 417 nucleotides, encoding a protein of 138 aa with 9 cysteine residues and a signal peptide located at the first 20 aa. The predicted molecular weight of full-length chIL-9 is 15718.41 Dalton (Da) and 13.6 kD without signal peptide. In addition, there are four Asparagine (Asn) residues at positions of 2, 37, 79, 90 of the sequence, three of which are the potential N-glycosylation sites in the mature chIL-9.

**Figure 1:**
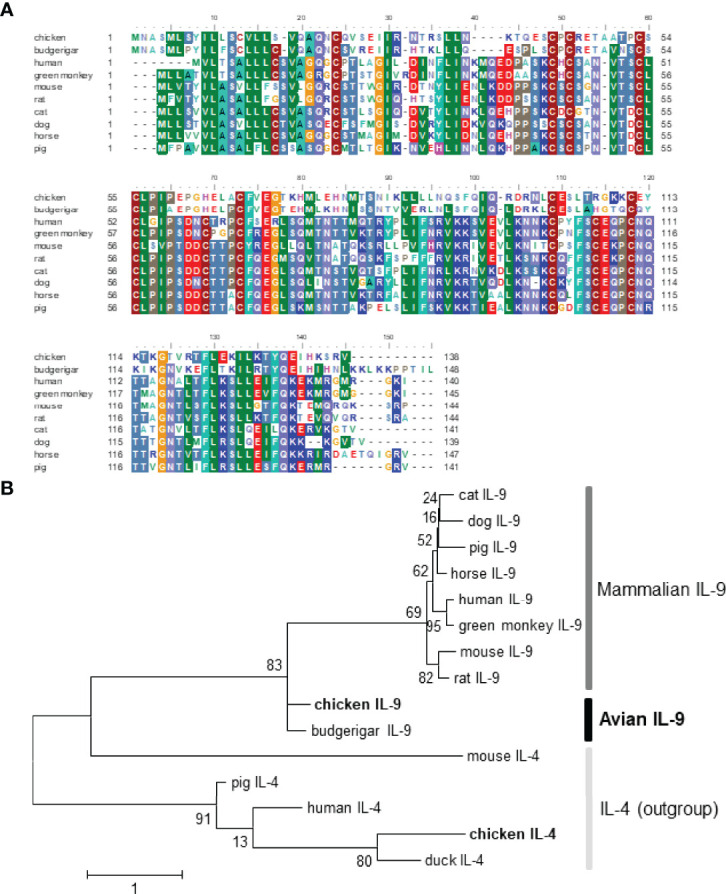
Sequence alignment and phylogenetic analysis of chicken IL-9 with the counterparts of other vertebrates. The GenBank accession numbers of IL-9 proteins are chosen as follow: chicken (*Gallus*; ACY79396.1), budgerigar (*Melopsittacus undulates*; XP_012984011.2), human (*Homo sapiens*; AAC17735.1), green monkey (*Chlorocebus sabaeus*; ACI28916.1), mouse (*Mus musculus*; NP_032399.1), rat (*Rattus norvegicus*; NP_001099217.1), cat (*Felis catus*; XP_003980942.1), dog (*Canis lupus familiaris*; XP_003431641.1), horse (*Equus caballus*; XP_001504404.1), pig (*Sus scrofa*; NP_001159515.1). The GenBank accession numbers of outgroup cytokine IL-4 include chicken (*Gallus*; CAF18427.1), duck (*Anas platyrhynchos*; ATP76495.1), human (*Homo sapiens*; NP_000580.1), mouse (*Mus musculus*; AAA39298.1), and pig (*Sus scrofa*; ADZ24282.1). **(A)** The sequence alignment of IL-9 was performed with BioEdit software with CLUSTAL W algorithm. **(B)** The phylogenetic analysis of chIL-9 and chIL-4 with other animals was performed with MEGA 6 software. The maximum likelihood tree was constructed and Bootstrap support values are shown for each node. The scale bar indicates the number of amino acid substitutions per site.

Compared with representative IL-9 sequences from budgerigar and mammals, chIL-9 displays 66.7% aa similarity to budgerigar but only has 26%-28% homology to mammalian counterparts without any conserved region (**Figure 1A** and **Table 2**). In addition, chIL-9 is 6 to10 aa shorter in length at the C-terminal of the protein sequence, compared to the IL-9s from budgerigar and other species (**Figure 1A**). Phylogenetic analysis indicated that chicken and budgerigar IL-9s formed a distinct cluster far away from mammalian IL-9s, reflecting the evolutionary relationship of this species to the others (**Figure 1B**). However, all the IL-9s were found to form a single evolutionary clade outside interleukin-4 (IL-4), another member of common γ-chain receptor cytokine family, suggesting that the IL-9s are orthologous (**Figure 1B**).

**Table 2:** Comparison of amino acid homology of chIL-9 with other animals.

Species	Homology (%)
budgerigar	66.7
human	27.7
Green monkey	27.6
mouse	26.1
rat	26.1
cat	27.8
dog	28.2
horse	27.4
pig	26.3

### The Abundance of chIL-9 mRNA in Tissues

**Figure 2:**
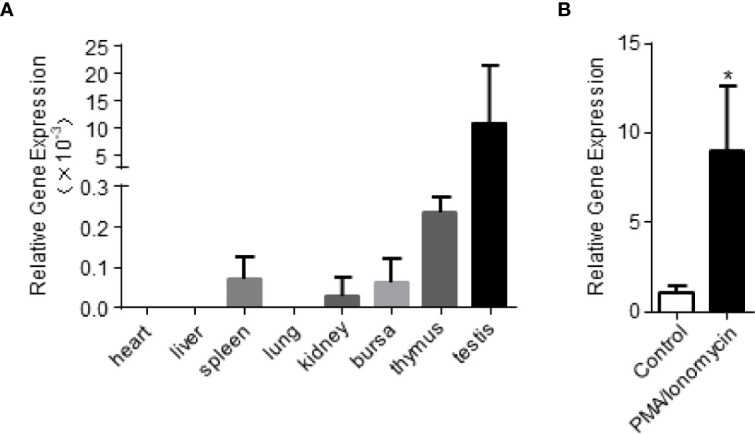
Quantitative RT-PCR analysis of chIL-9 mRNA expression in different tissues of chicken. **(A)** Total RNA was extracted from heart, liver, spleen, lung, kidney, bursa, thymus and testis of the chickens (n = 3), and then reverse transcribed into cDNA. The expression of chIL-9 mRNA was quantified by SYBR green-based real-time PCR assays. **(B)** Total RNA was extracted from non-activated or PMA/ionomycin-activated PBMCs of chickens (n = 3), and chIL-9 mRNA was quantified. Data shown are mean ± SD. *p < 0.05.

In order to examine the expression of chIL-9 in different tissues of chickens, we harvested heart, liver, spleen, lung, kidney, bursa, thymus and testis and quantify the abundance of chIL-9 mRNA by qPCR. We found that chIL-9 mRNA is expressed at highest level in the testis and lower level in the thymus, hardly detected in spleen, kidney, and bursa, and undetectable in heart, liver, and lung (**Figure 2A**). However, the expression of chIL-9 mRNA by PBMCs was significantly up-regulated upon stimulation with PMA/ionomycin (**Figure 2B**), suggesting that chicken has the potential to express IL-9 at cellular level though the tissue distribution of chIL-9 is restricted.

### Prokaryotic and Eukaryotic Expression of rchIL-9

**Figure 3:**
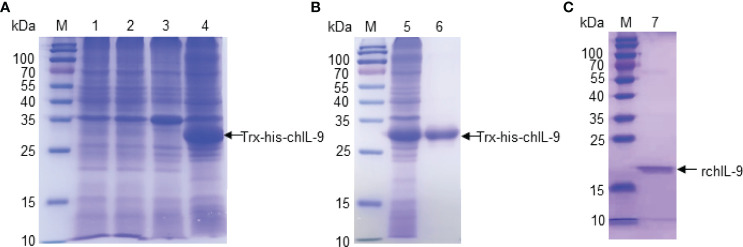
The expression and purification of recombinant chIL-9 in *E.coli*. Recombinant plasmid pET-32a-chIL-9 was transformed into *E.coli* and the recombinant bacteria were induced by IPTG for 4 h. Cell pellets were collected and lysed for analyzing the expression of recombinant chIL-9 and purification. **(A)** SDS-PAGE identified recombinant chIL-9 in the precipitate of bacterial lysate after induction by IPTG. M, Prestained Protein Ladder; lane 1 and 3, lysate supernatant and precipitate of uninduced bacteria; lane 2 and 4, IPTG-induced bacterial lysate supernatant and precipitate. **(B)** Recombinant chIL-9 was purified from the precipitate of bacterial lysate and confirmed by SDS-PAGE; **(C)** Recombinant chIL-9 without Trx-his tag was yielded.

In order to test the function of chIL-9, we firstly expressed recombinant chIL-9 in *E.coli* and DF-1 cells. The codons of ch*Il9* were optimized for *E.coli* and the signal peptide-deleted ch*Il9* was cloned into the pET-32a vector (**Figure S1A**). After confirmation by restriction enzymatic digestion (**Figure S1B**) and sequencing, recombinant chIL-9 (rchIL-9) was eventually expressed as a 32-kD fusion protein containing Trx-Tag, TEV cleavage site and his-Tag (Trx-his-chIL-9). SDS-PAGE showed that the rchIL-9 was largely expressed in a form of inclusion bodies after IPTG induction (**Figure 3A**). Then the protein was purified with Ni-NTA Resin and refolded, and subsequently subjected to cleavage by TEV protease (**Figure 3B**). An 18-kD rchIL-9 without Trx-his tag was yielded (**Figure 3C**).

**Figure 4:**
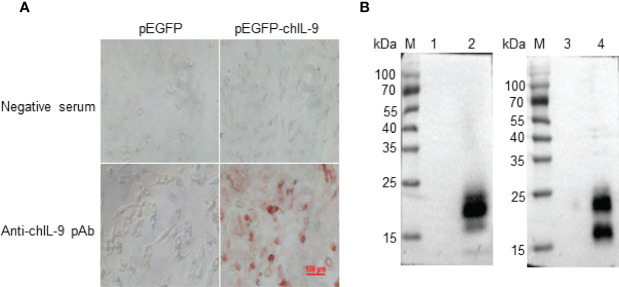
Recombinant chIL-9 was expressed in DF-1 cells. Recombinant plasmid pEGFP-chIL-9 was transfected into DF-1 cells and the expression of recombinant chIL-9 were detected by Immunocytochemistry (ICC) and Western Blot. **(A)** ICC showed that recombinant chIL-9 was expressed in pEGFP-chIL-9-transfected (right panel) but not pEGFP-transfected (left panel) DF-1 cells and recognized specifically by mouse anti-chIL-9 polyclonal antibodies (pAb) (lower panel). **(B)** Recombinant chIL-9 was detected by Western Blot in the whole cell lysate (left panel) and supernatant (right panel) of DF-1 cells that were transfected with pEGFP-chIL-9 plasmid (lane 2 and 4) but not pEGFP-C vector (lane 1 and 3).

In order to obtain eukaryotically-expressed rchIL-9, full-length ch*Il9* was amplified and inserted into pEGFP-C vector (pEGFP-chIL-9) (**Figure S1A**). After identification by sequencing and restriction enzymatic digestion (**Figure S1C**), the plasmid was purified and transfected into DF-1 cells and the expression of rchIL-9 was confirmed by Immunocytochemistry (ICC) and Western-blot (**Figure 4**). The results showed that rchIL-9 was successfully expressed in DF-1 cells (**Figure 4A**) and secreted into the culture supernatant (**Figure 4B**), which reacted specifically with anti-rchIL-9 pAb. Of note, the molecular weight of eukaryotically-expressed rchIL-9 is between 15 and 25 kD, one dominant band in the cell lysate and two bands in the supernatant (**Figure 4B**), larger than the theoretic size of mature chIL-9 (13.6 kD), implying that rchIL-9 expressed in DF-1 cells might be highly glycosylated.

### rchIL-9 Upregulates the Expression of Proinflammatory Cytokines by Chicken Monocytes/Macrophages

**Figure 5:**
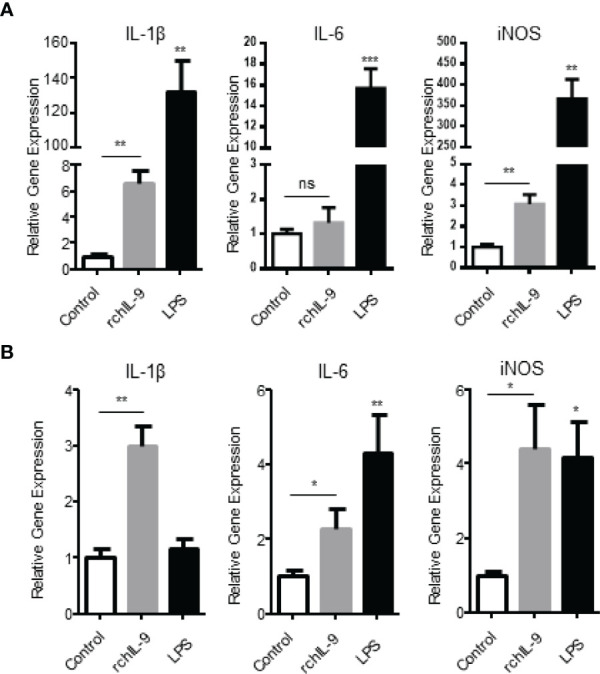
Recombinant chIL-9 upregulates the expression of proinflammatory cytokines by chicken monocytes/macrophages. Chicken macrophage cell line HD11 **(A)** and primary monocytes/macrophages **(B)** isolated from PBMCs were treated for 6 h with the supernatants from pEGFP-transfected (Control), pEGFP-chIL-9-transfected DF-1 cells (rchIL-9) at 1:1 dilution, and LPS (100 ng/mL), respectively. The cells were harvested and total RNA was extracted and reverse transcribed into cDNA. The expression of IL-1β, IL-6 and iNOS mRNAs was quantified by SYBR green-based real-time PCR assays. Data are presented as mean ± SD (n = 3) and representative of three independent experiments. ns, no significance, *p < 0.05, **p < 0.01, or ***p < 0.001.

Previous studies showed that murine IL-9 treatment activated BMDCs to produce more TNF-α, IL-1β, and IL-6 (8, 20), and monocytes and macrophages expressed high level of IL-9 receptor (21). Therefore, we tested the effect of rchIL-9 on chicken monocytes/macrophages. Chicken macrophage cell line HD11 and primary monocytes isolated from PBMCs were incubated with the supernatants from pEGFP-C- and pEGFP-chIL-9-transfected cells, respectively. Subsequently, the mRNA expressions of proinflammatory cytokines IL-1β, IL-6 and iNOS were quantified by RT-PCR. As shown in **Figure 5**, rchIL-9 treatment significantly upregulated the expression of IL-1β and iNOS mRNAs in HD11 cells (**Figure 5A**) and all three cytokines in primary monocytes (**Figure 5B**), compared to the treatment with the supernatant from pEGFP-C-transfected cells, though lower than the treatment of positive control LPS. These data suggest that eukaryotically-expressed rchIL-9 is biologically active in activating chicken monocytes/macrophages.

### rchIL-9 Induces Proliferation of Chicken CD3^+^ T Cells

**Figure 6:**
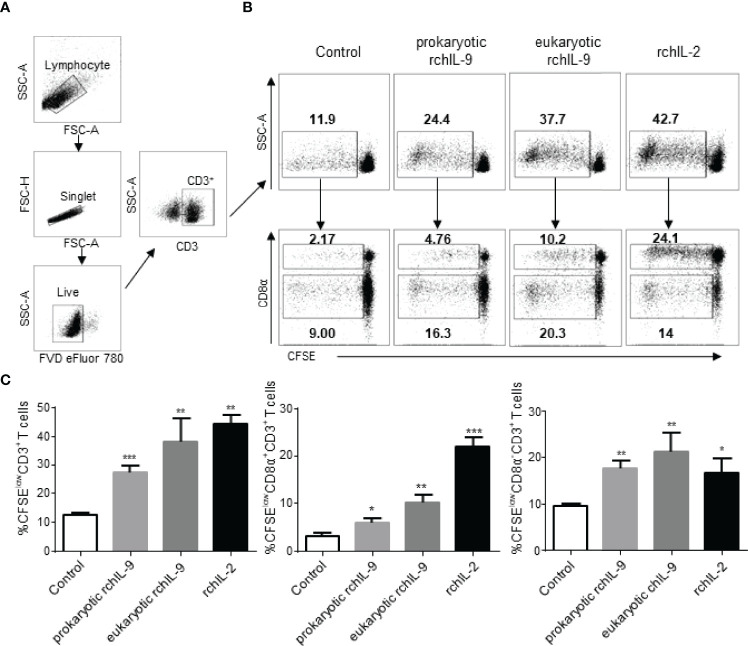
Recombinant chIL-9 induces proliferation of chicken CD3^+^ T cells. Chicken PBMCs (n = 3) were labelled with CFSE and then cultured for 5 days in the presence of the supernatant from mock transfected DF-1 cells (Control), pEGFP-chIL-9-transfected DF-1 cells (eukaryotic rchIL-9) at 1:1 dilution, purified rchIL-9 (100 ng/mL), and rchIL-2 (100 ng/mL), respectively. T cell proliferation was analyzed by flow cytometry. **(A)** Gating strategies to identify chicken T cells. Lymphocytes, single cells, live cells, and CD3^+^ or CD8α^+^ T-cell subsets were gated. **(B)** Representative dot-plots of proliferative total CD3^+^ T cells (upper panel), CD8α^+^CD3^+^ T cells and CD8α^-^CD3^+^ T cells (lower panel) were shown based on the reduction in CFSE fluorescence (CFSE^low^) between different groups. **(C)** The percentages of each proliferative T cells are compared between groups. Data are shown as mean ± SD. *p < 0.05, **p < 0.01, or ***p < 0.001.

As IL-9 was originally identified as a T cell growth factor, capable of inducing T cell proliferation (7). Therefore, we tested the effect of rchIL-9 on the proliferation of chicken T cells. Chicken PBMCs were labelled with CFSE and cultured in the presence of prokaryotically- and eukaryotically-expressed rchIL-9 as well as chicken IL-2. After culture for 5 days, T cell proliferation was examined by flow cytometry. As shown in **Figure 6**, compared to the control, both prokaryotically- and eukaryotically-expressed rchIL-9 as well as chicken IL-2 induced significant proliferation of CD3^+^ T cells. However, prokaryotically-expressed rchIL-9 is less effective than eukaryotically-expressed rchIL-9 and both rchIL-9s are less potent than chicken IL-2 (**Figures 6A, B**). Further analysis showed that rchIL-9 are more potent to induce CD4 T cells (CD3^+^ CD8α^-^) proliferation whereas chicken IL-2 are more effective to induce CD8 T cells (CD3^+^CD8α^+^) proliferation (**Figure 6C**). These findings suggest that rchIL-9 is biologically active and indeed capable of inducing T cells growth, with more effectiveness on CD4 T cells.

### Production and Characterization of Anti-chIL-9 mAbs

**Figure 7:**
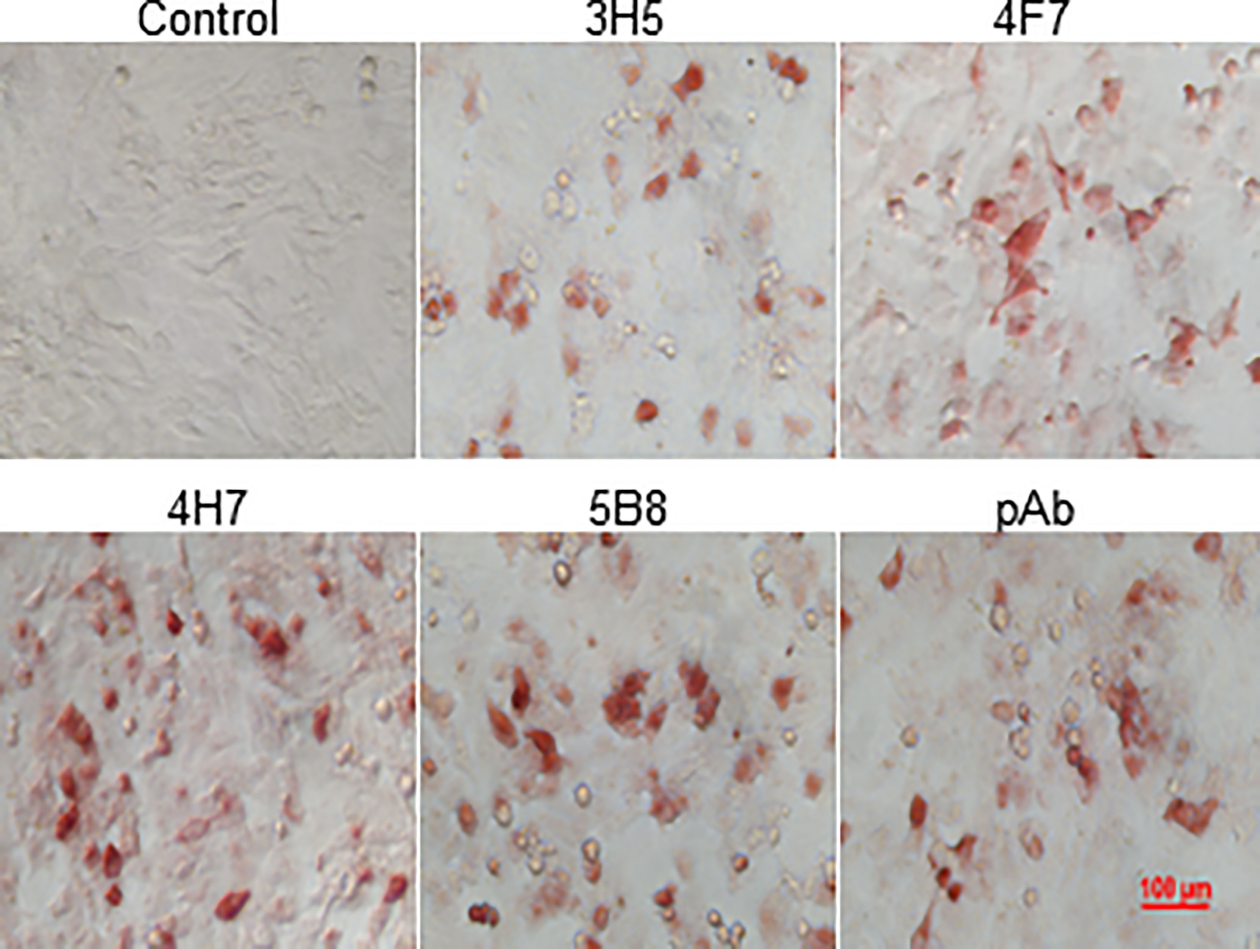
The reactivity of mAbs with recombinant chIL-9 expressed in DF-1 cells was identified by ICC. DF-1 cells were transfected with recombinant plasmid pEGFP-chIL-9 and cultured for 48 h and then fixed. The reactivity of four anti-chIL-9 mAbs (3H5, 4F7, 4H7 and 5B8), mouse negative serum (Control) and mouse anti-chIL-9 polyclonal antibodies (pAb) with chIL-9 expressed in DF-1 cells was examined by ICC. The images with magnification 400× are presented.

In order to identify natural chIL-9 protein expressed by chicken T cells, we firstly generated chIL-9-specific monoclonal antibodies (mAbs). Using prokaryotically-expressed rchIL-9 as immunogen, four mouse hybridoma cells that stably produce anti-chIL-9 mAb were obtained, namely 3H5, 4F7, 4H7 and 5B8. Ig subclass and isotyping showed that all the mAbs are IgG1 and κ chain (**Table 3**). Western blot showed that these mAbs specifically reacted with rchIL-9 but not with bacterial lysate or irrelevant Trx-tag-fused protein (**Figures S2A, B**). Further examination by ICC showed that all these mAbs, along with the mouse pAb, recognized chIL-9 expressed in pEGFP-chIL-9-transfected DF-1 cells (**Figure 7** and **Table 3**). These results suggest that rchIL-9 expressed in *E.coli* is immunogenic and these anti-chIL-9 mAbs are likely to recognize natural chIL-9.

**Table 3:** Characterization of anit-chIL-9 monoclonal antibodies by Immunocytochemistry and Western blotting.

mAb	Isotype	Ascites titre	ICC		Western blot	
			chIL-9	Trx-his-chIL-9	Irrelevant	*E. coli*
					Trx-his-chIL-7	
3H5	IgG1, κ	1: 1.64×10^6^	+ +	+	–	–
4F7	IgG1, κ	1: 4.10×10^5^	+ +	+	–	–
4H7	IgG1, κ	1: 3.28×10^6^	+ + +	+	–	–
5B8	IgG1, κ	1: 6.55×10^6^	+ +	+	–	–

ICC identified the binding ability of chIL-9 mAbs to eukaryotic rchIL-9, and Western Blot determined the reactivity of chIL-9 mAbs with prokaryotic rchIL-9 (Trx-his-chIL-9), irrelevant prokaryotic rchIL-7 (Trx-his-chIL-7) and *E.coli* proteins. -，no reaction, +，reaction, ++，moderate reaction, +++ strong reaction.

### Natural and Recombinant chIL-9 Is Potentially Glycosylated With a Molecular Weight of 25 kD

**Figure 8:**
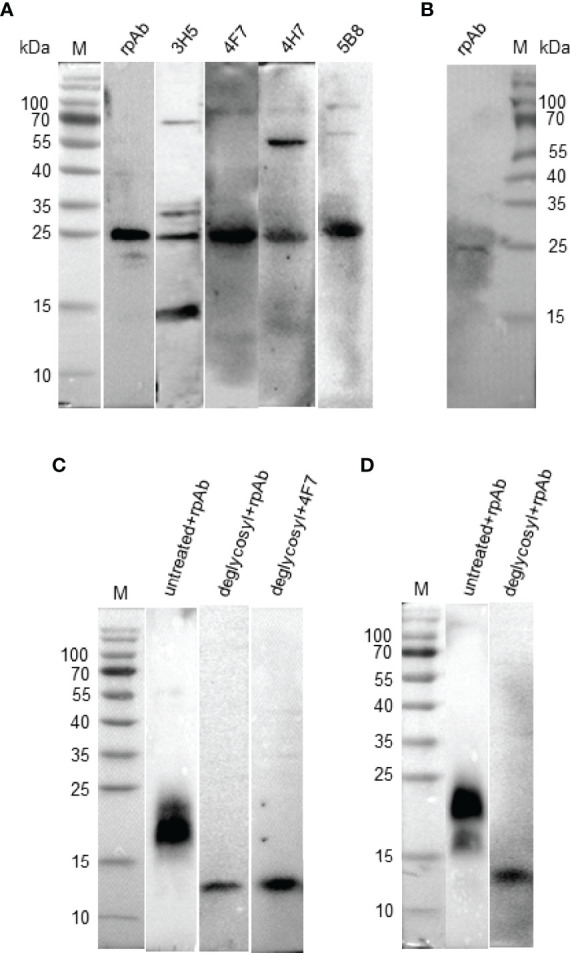
Natural and recombinant chIL-9 is potentially glycosylated with a molecular weight of 25 kD. PBMCs isolated from chicken were stimulated with PMA and Ionomycin in the presence or absence of brefeldin A for 4 h. The total protein was extracted from the activated PBMCs with RIPA lysis buffer **(A)** and the supernatant without brefeldin A inhibitor **(B)** was collected for SDS-PAGE and Western Blotting. Natural chIL-9 expressed by the activated chicken PBMCs was detected inside the cells **(A)** and in the supernatant **(B)** by four chIL-9 mAbs (3H5, 4F7, 4H7, and 5B8) and rabbit anti-chIL-9 pAb (rpAb), respectively. The whole cell lysates **(C)** and supernatant **(D)** of pEGFP-chIL-9-transfected DF-1 cells treated with or without deglycosylation enzyme was recognized with anti-chIL-9 pAb and mAb 4F7, showing a reduction of molecular weight.

IL-9 was reported to be a highly glycosylated protein in human and mouse, with a molecular weight between 32-39 kD (7). To confirm the size and expression form of natural chIL-9 protein, chIL-9 expressed by activated PBMCs was identified with anti-rchIL-9 mAbs and rabbit pAb by Western blotting. The results showed that rabbit anti-chIL-9 pAb and four mAbs all recognized a dominant band of 25 kD while mAb 3H5 and 4H7 also cross-reacted with 15-, 35-, 70- or 55-kD other bands (**Figure 8A**). Moreover, a single band of 25 kD was also identified in the supernatant of the activated PBMCs by rabbit anti-chIL-9 pAb (**Figure 8B**), implying a mature form of chIL-9 secreted by activated PBMCs. To alternatively confirm the glycosylation of chIL-9, the whole cell lysates and supernatant of pEGFP-chIL-9-transfected DF-1 cells were treated with deglycosylation enzyme (PNGase F). As a consequence, the molecular weight of rchIL-9 was reduced from about 20 kD to 13.6 kD (less than 15 kD) after deglycosylation (**Figures 8C, D**). These findings suggest that natural and mature chIL-9 expressed by activated PBMCs as well as rchIL-9 are potentially glycosylated while the unmodified chIL-9 is about 13.6 kD in theory.

## Discussion

As a member of the common γ-chain receptor cytokine family, IL-9 is an old cytokine. However, its diverse roles in autoimmune disorders, allergy, cancer and infectious diseases, various effects on both adaptive and innate immune cells and increasing discovery of its novel functions highlighted the uniqueness and importance of this cytokine in cytokine networks and immunomodulation (5, 11–15). Although mammalian IL-9s have been extensively investigated, avian IL-9 has not yet been characterized. In this study, through gene cloning and phylogenetic analysis, tissue distribution, recombinant expression, functional assays as well as the generation of anti-chIL-9 mAbs, we demonstrated for the first time the presence of functional IL-9 in chicken, a nonmammalian species.

IL-9 can be produced by a wide variety of immune cells and exerts various effects on both adaptive and innate immune cells (5). Indeed, we found that both prokaryotically and eukaryotically-expressed rchIL-9s are bioactive in inducing T cell proliferation and activating monocytes/macrophages *in vitro* (**Figures 5**, **6**), suggesting chIL-9 may have similar function to mammalian IL-9s. Of note, rchIL-9 preferentially promoted CD4 T cell proliferation (**Figure 6**), resembling the function of chicken IL-18 but different from chicken IL-2 that promotes CD8 T cell proliferation, which is consistent with a previous report (22) and the property of mammalian IL-9s (3). As for tissue distribution, IL-9 mRNA was found to be abundantly expressed in human brain and thymus, and at high levels in the testis and thymus of mouse and rat according to the RNA sequencing data in NCBI (Gene entry 3578 for human IL-9, 16198 for mouse IL-9 and 116558 for rat IL-9). Our data showed that chIL-9 mRNA was highly expressed in testis and thymus but not in other organs (**Figure 2A**), which is consistent with the expression pattern of IL-9 mRNA in mouse and rat as well as in human thymus. It is unclear why chIL-9 is more abundant in testis and thymus. Further investigation is needed to address the physiological role of chIL-9 and their cellular source in the testis and thymus.

Although rchIL-9 was produced in this study and functional *in vitro* for T cell proliferation and the activation of monocytes/macrophages, it is unclear what are the roles of chIL-9 *in vivo*. IL-9 was shown to be predominantly produced by a specialized T cell subset, Th9 (4). IL-9 and Th9 was found to have both anti-tumor and pro-tumor effect (10, 23). It would be interesting to explore whether Th9 is developed and differentiated in chickens and play roles in avian tumorigenic diseases like Marek’s disease and avian leukosis virus J subgroup. In addition, IL-9 have been shown to regulate T and B cell responses during respiratory syncytial virus infection (13), increase antigen-specific cytotoxic T lymphocyte response elicited by Food and mouth disease virus DNA vaccination (24), and play protective roles against Helicobacter pylori and parasitic worm infection (14, 15). These studies suggested IL-9 may have potential adjuvant effect for vaccine development. The availability of rchIL-9 and anti-chIL-9 mAbs from our study will help to test the *in vivo* function of chIL-9, identify the novel Th9 subset in the chicken and vaccine development for avian diseases.

In human and mouse, IL-9 is a highly glycosylated protein, with a molecular weight between 32-39 kD (7). However, in present study, we found that chIL-9 displays different size. Although the rchIL-9 produced in *E.coli* is signal peptide-truncated and Trx tag-cleaved, it has a molecular weight about 18 kD (**Figure 3C**), larger than the predicted molecular weight of 13.6 kD. Similarly, the eukaryotically-expressed rchIL-9 has two bands with molecular weight between 15 and 25 kD (**Figure 4B**) which is also more than the predicted 13.6 kD. However, the sizes of these rchIL-9 are less than 2 times of the predicted size 13.6 kD, suggesting that the rchIL-9 is more likely to be highly glycosylated but not polymerized. Indeed, glycosylation of rchIL-9 expressed in the transfected DF-1 cells was confirmed by the treatment with a deglycosylation enzyme (**Figures 8C, D**). Consistent with this fact, natural chIL-9 detected in the cell lysate and culture supernatant of activated PBMCs has a dominant band of 25 kD (**Figures 8A, B**), higher than the size of rchIL-9 expressed in the transfected DF-1 cells, indicating natural chIL-9 may undergo more glycosylated modifications after translation. Of note, mAb 3H5 and 4H7 may recognize off-target proteins with molecular weight higher or lower than 25 kD. Further analysis by mass spectrum in combination with immunoprecipitation may help to determine the expression form of natural chIL-9 in the future.

In summary, we demonstrated that chicken, a non-mammalian species, express functional IL-9, which is potentially a glycosylated protein with a molecular weight of 25kD. chIL-9 is evolutionarily conserved cluster different from mammal IL-9 and has restricted tissue distribution. Recombinant chIL-9 is biologically active in activating monocytes/macrophages and promoting CD3^+^ T cell proliferation. Anti-rchIL-9 mAbs and rabbit pAb recognized natural and mature chIL-9. This study lays the ground for further investigating the roles of chIL-9 in diseases and immunity.

## Data Availability Statement

The original contributions presented in the study are included in the article/**Supplementary Material**. Further inquiries can be directed to the corresponding author.

## Ethics Statement

All animal experiments were approved by Jiangsu Province Administrative Committee for Laboratory Animals (Permission number: SYXK-SU-2017-0007), and carried out in accordance with the guidelines of Jiangsu Province Laboratory Animal Welfare and ethics of Jiangsu Province Administrative Committee of Laboratory Animals.

## Author Contributions

SS and SH designed the experiment. SH, LC, XH, and YY carried out the experiments and analyzed the data. SS and SH wrote the manuscript. All authors contributed to the article and approved the submitted version.

## Funding

This study is funded by National Natural Science Foundation of China (32002293) and A Project Funded by the Priority Academic Program Development of Jiangsu Higher Education Institutions (PAPD).

## Conflict of Interest

The authors declare that the research was conducted in the absence of any commercial or financial relationships that could be construed as a potential conflict of interest.

## Publisher’s Note

All claims expressed in this article are solely those of the authors and do not necessarily represent those of their affiliated organizations, or those of the publisher, the editors and the reviewers. Any product that may be evaluated in this article, or claim that may be made by its manufacturer, is not guaranteed or endorsed by the publisher.

## Supplementary Material

Supplementary Figure 1 Cloning and construction of recombinant plasmids pET32a-chIL-9 and pEGFP-chIL-9. The signal peptide-deleted (354 nt) and full-length chicken *Il9* (417 nt) gene were cloned into pET32a and pEGFP-C vector **(A)**, respectively. Then the constructs were confirmed by restriction enzymatic digestion **(B)** and **(C)**. M, DNA ladder; lane 1, double digestion product of pET32a-chIL-9; lane 2, double digestion product of pEGFP-chIL-9.

Supplementary Figure 2 The reactivity of mAbs with recombinant chIL-9 identified by Western blot. **(A)** Representative SDS-PAGE of *E. coli* lysate (lane 1), purified rchIL-9 (lane 2), irrelevant Trx-his-chIL-7 protein (lane 3). **(B)** Western blot analysis of the specificity of four anti-chIL-9 mAbs to prokaryotic rchIL-9. M, Pre-stained protein ladder; lane 1, *E. coli* lysate; lane 2, purified rchIL-9; lane 3, irrelevant Trx-his-chIL-7 protein.
