# Supplementary material for: First Characterization of Chicken Interleukin-9
Source: Front Immunol. 2022 Jun 20;13:889991. doi: 10.3389/fimmu.2022.889991 (PMC9252340; doi:10.3389/fimmu.2022.889991)
Supplement: Supplementary Figure 1 — Cloning and construction of recombinant plasmids pET32a-chIL-9 and pEGFP-chIL-9. The signal peptide-deleted (354 nt) and full-length chicken Il9 (417 nt) gene were cloned into pET32a and pEGFP-C vector (A), respectively. Then the constructs were confirmed by restriction enzymatic digestion (B) and (C). M, DNA ladder; lane 1, double digestion product of pET32a-chIL-9; lane 2, double digestion product of pEGFP-chIL-9. [file Presentation_1.pptx]

## Slide 1
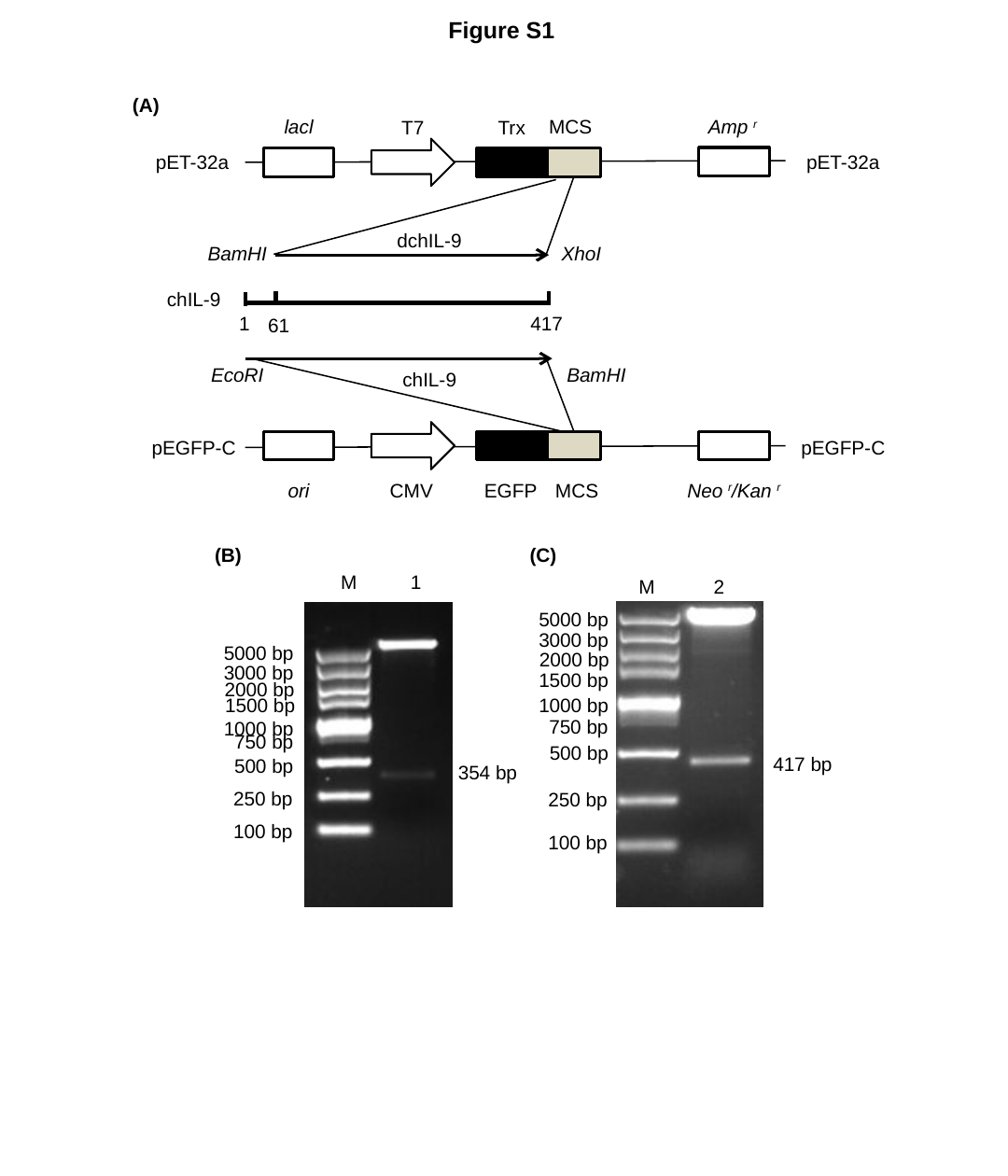

Figure S1
(A)
Amp r
MCS
lacl
Trx
T7
pET-32a
pET-32a
dchIL-9
XhoI
BamHI
chIL-9
417
61
EcoRI
BamHI
chIL-9
pEGFP-C
pEGFP-C
Neo r/Kan r
ori
CMV
MCS
EGFP
(B)
(C)
 M 1
354 bp
5000 bp
3000 bp
2000 bp
1500 bp
1000 bp
750 bp
500 bp
250 bp
100 bp
 M 2
417 bp
5000 bp
3000 bp
2000 bp
1500 bp
1000 bp
750 bp
500 bp
250 bp
100 bp
1

## Slide 2
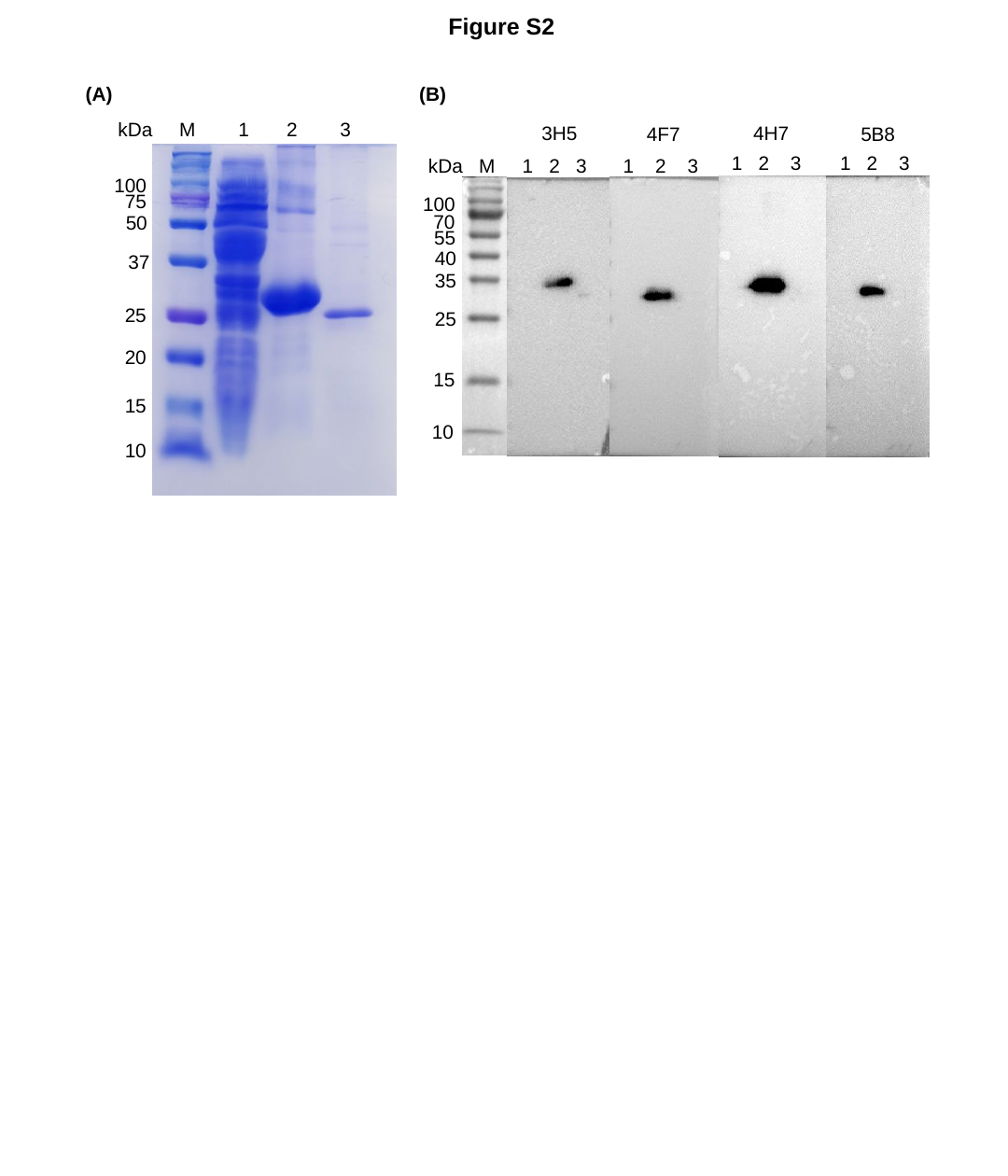

Figure S2
(A)
(B)
kDa M 1 2 3
100
75
 50
37
25
20
15
10
3H5
4H7
4F7
5B8
1 2 3
1 2 3
 kDa M
 1 2 3
1 2 3
100
70
 55
40
35
25
15
10
